# Demographic Predictors of Outpatient Mental Health Service Utilization in the United States

**DOI:** 10.7759/cureus.90683

**Published:** 2025-08-21

**Authors:** Akintunde C Akinboboye, Oluwanifesimi D Olu-Lawal, Sunday O Arifayan, Chinonso F Eziechi, Echezonachukwu S Eziechi, Stanley Ezulike, Okelue E Okobi

**Affiliations:** 1 Emergency Medicine, University of Medical Sciences Teaching Hospital, Ondo, NGA; 2 Psychiatry and Behavioral Sciences, Jesse Brown Veterans Affairs Medical Center, Chicago, USA; 3 College of Health Sciences, University of Ilorin Teaching Hospital, Ilorin, NGA; 4 Public Health, Liberty University, Lynchburg, USA; 5 Psychiatry, Maitama District Hospital, Abuja, NGA; 6 Internal Medicine, Chukwuemeka Odumegwu Ojukwu University Teaching Hospital, Awka, NGA; 7 Family Medicine, Larkin Community Hospital Palm Springs Campus, Miami, USA; 8 Family Medicine, IMG Research Academy and Consulting LLC, Homestead, USA

**Keywords:** healthcare disparities, health services utilization, mental health services, outpatients, sociodemographic factors, united states

## Abstract

Background

Mental health disorders represent a growing public health concern in the United States, yet disparities in outpatient service utilization persist across demographic groups. Understanding these disparities is essential for targeted intervention and equitable resource allocation. This study aimed to identify independent demographic predictors of outpatient mental health service utilization using nationally representative data.

Methodology

We conducted a retrospective cross-sectional analysis using data from the National Hospital Ambulatory Medical Care Survey, aggregating outpatient visits from 2005 to 2011. Adults aged 18 and older were included. Mental health-related visits were identified using the International Classification of Diseases diagnostic codes and reason-for-visit criteria. Multivariate survey-weighted logistic regression was used to examine associations between service utilization and demographic variables, i.e., age, sex, race/ethnicity, and insurance status.

Results

Of the 134,500 adult outpatient visits analyzed, 1,469 visits (9.2% of 15,388 weighted visits) were classified as mental health-related. Adjusted analyses showed that Hispanic individuals (odds ratio (OR) = 0.056, 95% confidence interval (CI) = 0.012-0.260) and those of Other race/ethnicity (OR = 0.062, 95% CI = 0.001-0.484) had significantly lower odds of receiving mental health services compared to non-Hispanic Whites. Sex, age, and insurance type were not statistically significant predictors.

Conclusions

Racial and ethnic disparities remain a key barrier to equitable outpatient mental health care in the United States. Culturally tailored interventions are needed to improve access and utilization among underserved populations.

## Introduction

Recently, mental health disorders have become a serious public health issue in the United States, as the estimated number of people affected by mental illness annually is increasing [1]. The National Institute of Mental Health explains that almost one in five U.S. adults was diagnosed with a mental disorder, such as depression, anxiety, bipolar disorder, and schizophrenia, which have become the most frequent psychological issues [2,3]. The ability to receive proper mental healthcare (especially on an outpatient basis) promptly is also an essential concept to the successful treatment and enhanced patient outcomes [4]. Psychiatric assessment, psychotherapy, drug management, and follow-ups to outpatient mental health services are the mainstay of mental healthcare among Americans [5,6]. Although stigmatization is becoming less of an issue, there still exist differences in mental health service utilization, which, in many cases, is even stimulated by the diversity of demographic and socioeconomic factors [7].

Age, race/ethnicity, sex, and insurance membership are demographic factors that are significant determinants of the propensity of an individual to get mental healthcare [8]. For example, research has also indicated continuously that racial and ethnic minorities are less likely to utilize mental health services than non-Hispanic Whites, despite an adjustment for the presence of mental health need [9-11]. Age and sex also have a key influence on the mental health patterns in providing services. Similarly, patterns by age and sex have been described; younger adults often show greater help-seeking and awareness of mental health issues, whereas older adults may face generational barriers and reduced access, and women frequently demonstrate higher utilization for some types of mental healthcare [12-14].

Despite this growing literature, gaps remain in understanding which demographic factors independently predict outpatient mental health service utilization at the national level. Much evidence to date is derived from localized samples or specific subpopulations, limiting generalizability [15-17]. Nationally representative data, such as the National Hospital Ambulatory Medical Care Survey (NHAMCS), provide an opportunity to examine outpatient visit-level utilization patterns across the U.S. population [18].

Such predictors will carry essential information to guide policy levels of intervention, allocation of resources, and formulation of specific interventions that can raise equity in mental healthcare provision. Knowledge of which groups are at risk of not receiving outpatient mental health services or are more likely to receive outpatient mental health services is essential. It will assist clinicians, health systems, and policymakers in planning to reduce inequalities and properly orient resources to meet the unmet mental health needs [17,19]. The outcome will lead to a larger objective of a more inclusive and accessible mental healthcare system in the United States and, consequently, to a more effective one. Therefore, this study aims to determine whether age, race/ethnicity, sex, and insurance status independently predict the likelihood of outpatient mental health visits among U.S. adults using NHAMCS outpatient department data from 2005 to 2011 via multivariate survey-weighted logistic regression.

## Materials and methods

Study design and data source

This study employed a retrospective cross-sectional design using data from NHAMCS, a nationally representative survey conducted annually by the National Center for Health Statistics, a division of the Centers for Disease Control and Prevention [20]. The NHAMCS utilizes a multistage probability sampling method to collect data from visits to hospital outpatient departments and emergency departments across the United States. It includes detailed information on patient demographics, reasons for visit, diagnoses, services rendered, and payment sources. For this analysis, data from 2005 to 2011 were aggregated to enhance statistical power and capture national trends over time. These years were selected because the outpatient department component of the NHAMCS provided consistent, complete sampling frames and variable definitions across 2005-2011; after 2011, the NHAMCS sampling emphasis and available variables for the outpatient department changed, limiting comparability with later cycles. Aggregating multiple years increases sample size and precision for subgroup analyses (for example, by race/ethnicity and age categories) but may reduce sensitivity to short-term year-to-year changes; accordingly, findings are presented as pooled estimates representative of the 2005-2011 period rather than as year-specific trends. The NHAMCS dataset is de-identified and includes survey weights, strata, and primary sampling units, which allow for accurate estimation of national utilization patterns in healthcare services, including mental healthcare. Because of the complex survey design, all analyses accounted for survey weights and design variables to obtain unbiased point estimates and correct variance estimates for inference to the U.S. outpatient visit population. When pooling multiple survey years, the annual visit-level weights were adjusted for pooling by dividing each year’s visit weight by the number of years pooled so that estimates represent the average annual visit experience across 2005-2011.

Study population

The study population included all patient visits recorded in the outpatient department component of the NHAMCS during the 2005-2011 period. To identify outpatient mental health visits, we used a combined algorithm based on diagnosis and reason-for-visit codes. Specifically, diagnosis codes included mental disorder ranges and selected specific International Classification of Diseases, Ninth Revision, Clinical Modification (ICD-9-CM) codes (for clinic years using ICD-9): ICD-9-CM codes 290-319 (mental disorders) with primary emphasis on schizophrenia spectrum and other psychotic disorders (295.), mood disorders (296. and 311), anxiety and somatoform diagnoses (300. and 300.2), adjustment disorders (309.), and substance-related disorders (291., 292., 303., 304., 305.). In years or records coded with International Classification of Diseases, 10th Revision (ICD-10) equivalents, mapped ICD-10-CM codes for the same diagnostic groups were used. In addition to diagnosis codes, visits were classified as mental health-related when reason-for-visit or procedure codes indicated psychiatric evaluation, psychotherapy, counseling, or behavioral health follow-up. Visits with missing key demographic data (age, sex, race/ethnicity, or insurance) were excluded from the primary analytic sample using a complete-case approach. The final analytic sample was restricted to adults aged 18 years and older to ensure focus on independent utilization behavior in the adult population.

Variables

The primary outcome variable was whether or not an outpatient visit was for mental health services, categorized as a binary variable (mental health-related visit vs. non-mental health visit). This determination was made based on either a mental health diagnosis or a mental health-related reason for the visit. The primary independent variables of interest were key demographic characteristics, including age, sex, race/ethnicity, and insurance status. Age was categorized into the following five groups: 18-24, 25-34, 35-49, 50-64, and 65 years and older. Race and ethnicity were constructed from NHAMCS race and Hispanic-ethnicity fields and collapsed into the following four mutually exclusive categories: Non-Hispanic White, Non-Hispanic Black, Hispanic (of any race), and Other (including Asian, Native American/Alaska Native, multiracial, and other small groups). Sex was coded as recorded in the NHAMCS file (male or female). Insurance status was derived from the primary expected source of payment and classified into the following four categories: private insurance, Medicare, Medicaid, and uninsured/self-pay. Geographic region and visit urgency were coded using the NHAMCS standard variables but were not included in the primary model unless they met the criteria for inclusion in preliminary analyses. Additional covariates such as geographic region and visit urgency were considered but not included in the final model unless found to be significant in preliminary analyses.

Statistical analysis

Descriptive statistics were first computed to characterize the sample population by demographic variables and to compare mental health-related visits with non-mental health visits. Because the data derive from a complex survey design, design-based F-tests (survey-adjusted Rao-Scott F-tests) were used to assess univariate associations between categorical predictors and mental health service utilization. Multivariate survey-weighted logistic regression was then performed to identify independent demographic predictors of outpatient mental health service use. Logistic regression was chosen because the outcome is binary (mental health-related visit vs. not) and the method permits estimation of adjusted odds ratios (aORs) while controlling for multiple covariates simultaneously. The dependent variable in the model was a binary indicator of whether a visit was for mental healthcare. Independent variables included age group, sex, race/ethnicity, and insurance type. All models accounted for the NHAMCS complex sampling design by incorporating survey weights, strata, and primary sampling units using Stata 18 survey commands to produce nationally representative estimates and obtain correct standard errors. aORs and 95% confidence intervals (CIs) were reported. Statistical significance was set at a two-sided p-value of less than 0.05. Model diagnostics included assessment of multicollinearity using variance inflation factors (VIFs) for the key predictors and inspection of standard errors; where indicated, categorical reference groups were chosen to avoid sparse-category instability. Goodness-of-fit and model adequacy were evaluated using survey-appropriate diagnostics (for example, survey-adjusted Wald tests and examination of model residuals), recognizing the limitations of some traditional fit statistics in complex-survey contexts. All analyses were performed using Stata version 18 (StataCorp, College Station, TX, USA), using survey commands to properly account for sampling weights, strata, and primary sampling units.

Ethical considerations

This study used de-identified, publicly available data from the NHAMCS, which poses no risk to individual participants. As such, the analysis qualified for exemption from institutional review board approval under federal guidelines. No direct patient interaction occurred, and no protected health information was accessed. All data handling and reporting adhered to ethical standards for research involving secondary analysis of publicly available datasets.

## Results

Table 1 presents the weighted distribution of outpatient visits by key demographic characteristics, stratified by whether the visit was mental health-related. Frequencies and weighted percentages are reported for sex, age group, insurance status, and race/ethnicity, with comparisons tested using design-based F-tests to account for the complex sampling structure of the NHAMCS dataset. The analysis included 134,500 adult outpatient visits. These descriptive comparisons provide foundational insight into the unadjusted relationships between demographic characteristics and mental health service utilization. Statistically significant differences were observed across age groups (p = 0.04) and race/ethnicity (p < 0.001), suggesting variation in mental health-related service use among demographic subgroups.

**Table 1:** Distribution of mental health-related outpatient visits by demographic characteristics in the United States (NHAMCS, 2005–2011). Values are presented as weighted frequencies (N) with weighted column percentages (%). Design-based F-tests were used for group comparisons. Statistical significance level: *: p < 0.05, **: p < 0.01, ***: p < 0.001. NHAMCS: National Hospital Ambulatory Medical Care Survey

Variable	Mental health-related visit (N = 1,469)	Mental health-unrelated visit (N = 13,919)	Design-based F-test	P-value
Sex, %			1.31	0.25
Male	1,054 (11%)	8,815 (89%)		
Female	415 (8%)	5,104 (92%)		
Age group (in years), %			2.53	0.04*
18–24	109 (8%)	1,225 (92%)		
25–34	231 (11%)	1,911 (89%)		
35–49	545 (15%)	3,186 (85%)		
50–64	475 (11%)	3,827 (89%)		
65+	109 (3%)	3,770 (97%)		
Insurance (primary expected source of payment), %			2.08	0.11
Private	690 (12%)	5,029 (88%)		
Medicare	153 (4%)	3,749 (96%)		
Medicaid	238 (7%)	3,060 (93%)		
Uninsured	148 (9%)	1,456 (91%)		
Race/Ethnicity, %			7.19	<0.001
Non-Hispanic White	1,360 (12%)	10,124 (88%)		
Non-Hispanic Black	80 (4%)	2,049 (96%)		
Hispanic	27 (2%)	1,374 (98%)		
Other	2 (1%)	372 (99%)		

Among outpatient visits captured between 2005 and 2011, differences in mental health service utilization were observed across age groups and racial/ethnic categories, while sex and insurance status did not show statistically significant variation. Mental health-related visits were slightly more common among male visits, 1,054 (11%), compared to female visits, 415 (8%), although this difference was not statistically significant (F = 1.31, p = 0.25). Overall, the descriptive results indicate that disparities are most pronounced by race/ethnicity, with age showing more modest variation concentrated among middle-aged groups, while sex and payer show little unadjusted difference; this pattern orients the reader to the primary axes of disparity that informed multivariable modeling.

Age was significantly associated with mental health-related visits (F = 2.53, p = 0.04). The highest proportion of mental health visits occurred among patients aged 35-49 years, 545 (15%) visits, followed by those aged 25-34 years, 231 (11%) visits, and 50-64 years, 475 (11%) visits. Younger adults aged 18-24 years reported 109 (8%) visits for mental healthcare, whereas individuals aged 65 and older had the lowest proportion, with only 109 (3%) visits related to mental health, suggesting a steep decline in utilization among the elderly.

**Figure 1:**
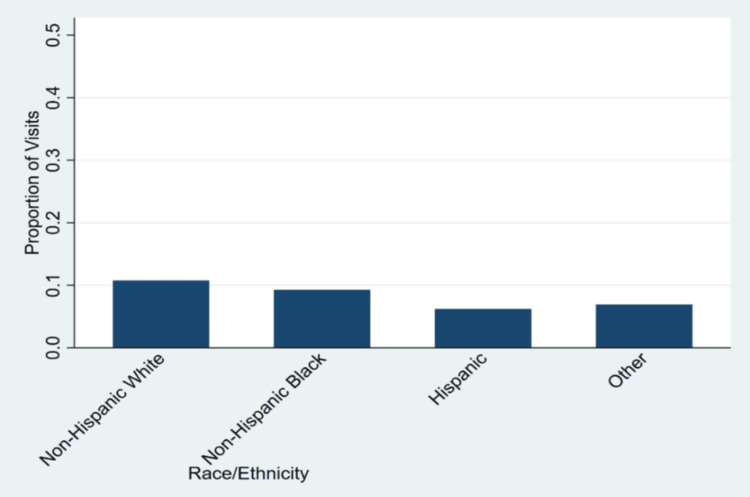
Proportion of outpatient mental health-related visits by race/ethnicity, United States (NHAMCS 2005–2011). This figure displays the weighted percentage (%) of outpatient visits that were mental health-related, stratified by race/ethnicity. Proportions are based on survey-weighted estimates using the NHAMCS complex sampling design. Statistical significance level: *: p < 0.05, **: p < 0.01, ***: p < 0.001. NHAMCS: National Hospital Ambulatory Medical Care Survey

The primary expected source of payment was not significantly associated with mental health visit status (F = 2.08, p = 0.11). Visits paid through private insurance accounted for the largest share of mental health-related encounters, 690 (12%) visits, followed by Medicaid, 238 (7%) visits, uninsured/self-pay, 148 (9%) visits, and Medicare, 153 (4%) visits. In contrast, race and ethnicity showed a strong and statistically significant association with mental health-related visits (F = 7.19, p < 0.001). Non-Hispanic White patients had the highest proportion of mental health-related visits, 1,360 (12%) visits, followed by Non-Hispanic Black patients, 80 (4%) visits. Hispanic individuals recorded only 27 (2%) visits for mental health issues, while those categorized as “Other” racial/ethnic groups had the lowest proportion, with just 2 (1%) visits. Estimates for the “Other” group are likely unstable and confidence intervals correspondingly wide; this limitation is considered in subsequent multivariable analyses and the Limitations section. These disparities are further visualized in Figure 1, which illustrates the proportion of mental health-related visits across racial and ethnic groups.

Table 2 indicates the multivariate survey-weighted logistic regression predicting outpatient mental health visits.

**Table 2:** Multivariate survey-weighted logistic regression predicting outpatient mental health visits. Reference categories: Male (sex), 18–24 (age), Non-Hispanic White (race/ethnicity), Private insurance (insurance). Adjusted odds ratios (aORs) with 95% confidence intervals (CIs) are presented from multivariate survey-weighted logistic regression models. Significance level: *: p < 0.05, **: p < 0.01, ***: p < 0.001.

Predictor	Odds ratios	95% confidence intervals
Sex		
Female	0.549	(0.248, 1.216)
Age Group (reference: 18–24)		
25–34	1.206	(0.311, 4.672)
35–49	1.918	(0.549, 6.695)
50–64	1.034	(0.245, 4.365)
65+	0.349	(0.060, 2.044)
Insurance (reference: Private)		
Medicare	0.689	(0.179, 2.652)
Medicaid	0.790	(0.269, 2.320)
Uninsured	0.874	(0.312, 2.446)
Race/Ethnicity (reference: Non-Hispanic White)		
Non-Hispanic Black	0.276	(0.070, 1.082)
Hispanic	0.056^***^	(0.012, 0.260)
Other	0.062^**^	(0.001, 0.484)

After adjusting for all demographic variables in the model, race and ethnicity emerged as the strongest independent predictors of outpatient mental health service utilization. Compared to non-Hispanic White patients, Hispanic individuals had significantly lower odds of receiving mental health-related outpatient care (aOR = 0.056, 95% CI = 0.012-0.260, p < 0.001), as did patients in the “Other” racial/ethnic category (aOR = 0.062, 95% CI = 0.001-0.484, p < 0.01). Although the OR for non-Hispanic Black patients was also below 1.0 (aOR = 0.276), the confidence interval (95% CI = 0.070-1.082) included the null value, indicating the result was not statistically significant at the conventional level.

No statistically significant differences in outpatient mental health visit likelihood were observed by sex, though females had lower odds compared to males (aOR = 0.549, 95% CI = 0.248-1.216). Likewise, age was not a significant predictor across any category. Compared to young adults aged 18-24 years, individuals aged 35-49 years had the highest odds (aOR = 1.918, 95% CI = 0.549-6.695), but wide CIs indicate limited precision and lack of statistical significance. Patients aged 65 and older had the lowest odds of receiving outpatient mental health services relative to the youngest group (aOR = 0.349, 95% CI = 0.060-2.044), though this too was not statistically significant.

**Figure 2:**
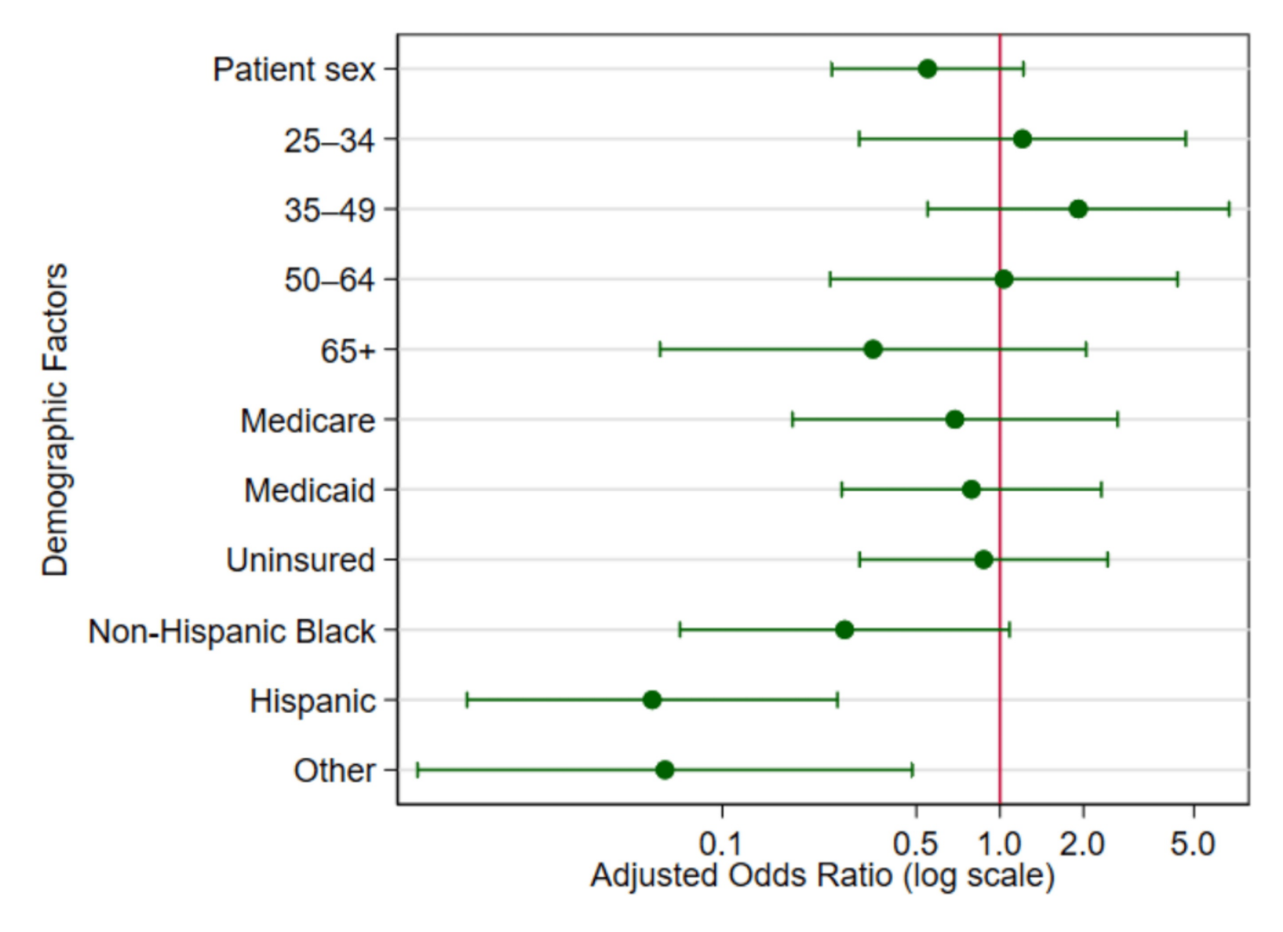
Adjusted odds ratios of demographic predictors for outpatient mental health-related visits (NHAMCS 2005–2011). Forest plot showing adjusted odds ratios and 95% confidence intervals for sex, age, insurance type, and race/ethnicity. The vertical reference line represents an odds ratio of 1.0. All estimates are derived from multivariate survey design logistic regression models adjusted for all other listed covariates. NHAMCS: National Hospital Ambulatory Medical Care Survey

Insurance status also did not significantly predict outpatient mental health utilization. Compared to privately insured individuals, those with Medicare (aOR = 0.689, 95% CI = 0.179-2.652), Medicaid (aOR = 0.790, 95% CI = 0.269-2.320), or no insurance (aOR = 0.874, 95% CI = 0.312-2.446) showed lower odds, but none of these associations reached statistical significance. These findings are further presented in Figure 2.

As illustrated in Figure 2, most demographic predictors had adjusted ORs close to or below 1.0, reflecting generally reduced odds of outpatient mental health utilization relative to reference groups. The strongest disparities were seen among racial/ethnic categories, where Hispanic and “Other” race groups had markedly lower odds of mental health-related visits, with their CIs not crossing the null value, indicating statistically significant effects. These findings are consistent with those reported in Table 2. Notably, other variables, including age, sex, and insurance status, showed wider confidence intervals and no significant departures from the reference category, reinforcing the observation that race and ethnicity were the primary demographic predictors of outpatient mental healthcare utilization in this model.

## Discussion

This study used a nationally representative sample from the NHAMCS (2005-2011) to identify demographic predictors of outpatient mental health service utilization in the United States. The results highlight significant disparities in utilization patterns, particularly by race and ethnicity, while no significant associations were found for sex, insurance status, or most age groups.

Notably, the most prominent finding was the significantly lower likelihood of mental health-related outpatient visits among Hispanic and “Other” racial/ethnic groups, even after adjusting for sex, age, and insurance status. Hispanic individuals had 94% lower odds of receiving outpatient mental healthcare compared to non-Hispanic Whites, while individuals classified as “Other” had 93% lower odds. These results align with prior studies reporting persistent underutilization of mental health services among racial and ethnic minority populations despite comparable or even greater mental health needs [10-13]. Structural barriers, including limited access to culturally competent care, language differences, stigma, and lack of trust in healthcare systems, may contribute to this pattern [7,15].

Hispanic individuals and those in the category of “Other” racial/ethnic groups underutilize mental health services. There are several possible reasons for this, including language and cultural barriers, stigma surrounding mental health, and lack of knowledge about available mental health services. There are some ways to overcome these barriers to mental healthcare for this specific population. Expanding the availability of interpreters and language-concordant providers addresses the language barrier. Training clinicians in cultural competence can reduce cultural impediments. Also, increasing community outreach and education can be helpful [15].

Although non-Hispanic Black individuals also showed lower odds of outpatient mental health visits, the association did not reach statistical significance. This may reflect variability within subgroups or be due to sample size limitations, even though the trend is consistent with previous research showing that Black Americans are less likely to seek outpatient mental healthcare even when services are available [11]. Further, contrary to expectations and previous literature, no significant differences in utilization were observed across insurance categories or between sexes. While females were slightly less likely than males to receive outpatient mental health services in this sample, the association was not statistically significant. This finding diverges from prior studies that typically report higher utilization among women [14] and may reflect changing patterns in mental health awareness, care-seeking behaviors, or access among men in recent years. Similarly, the lack of association between insurance type and mental health utilization may suggest that barriers to care extend beyond insurance coverage alone and involve factors such as provider availability, regional access disparities, and socioeconomic status variables not directly captured in this study.

Still, age did not emerge as a significant predictor, although adults aged 35-49 years had the highest odds of utilizing outpatient mental healthcare, while adults aged 65 and older had the lowest. This is consistent with earlier research indicating that older adults often face generational stigma, reduced mental health literacy, and limited access to mental health professionals, particularly those trained in geriatric psychiatry [12].

These findings have important implications for public health policy and clinical practice. The evident racial and ethnic disparities suggest an urgent need to improve access to and the cultural relevance of mental health services for minority populations. Initiatives such as expanding language-concordant providers, training clinicians in cultural competence, and increasing community outreach and education may help address some of the underlying causes of inequitable care. Additionally, broadening access alone may not be sufficient if services are not tailored to the unique needs and preferences of underserved groups.

Study limitations

This study has several limitations. First, the analysis was based on NHAMCS data from 2005 to 2011, which may not fully reflect current trends in mental health service utilization. This time frame was selected due to data availability and completeness for outpatient department visits during those years. In more recent years, NHAMCS shifted focus to emergency department data, limiting access to nationally representative outpatient mental health data for adult populations.

Second, as with all cross-sectional survey designs, causal relationships cannot be inferred from these findings. The analysis captures visit-level data rather than individual-level behavior, meaning multiple visits from the same person may be represented; this can bias estimates if frequent users differ systematically from single-visit users. Third, certain potentially important variables, such as income, education, mental health severity, geographic location, and provider availability, were not included in the dataset, which may limit the comprehensiveness of the model. Additionally, reliance on administrative coding may lead to underreporting or misclassification of mental health visits (for example, visits with primary somatic complaints but underlying psychiatric needs may be missed). The transition between diagnostic coding systems across years (ICD-9 to ICD-10 in some data contexts) and differences in how reason-for-visit codes are recorded may also contribute to misclassification or heterogeneity across cycles.

Other important limitations include the small, unweighted cell counts for some subgroups (notably the “Other” race/ethnicity category with very few mental health-related visits), which can produce unstable estimates and wide CIs in both descriptive and multivariable analyses. Excluding visits with missing demographic data may have introduced selection bias if excluded visits differed systematically in their likelihood of being mental health-related. Residual confounding is possible because unmeasured or imperfectly measured factors (for example, severity of illness, health literacy, language proficiency, and social determinants such as housing instability) may influence both demographic group membership and service utilization. Although survey weights, strata, and primary sampling units were used to produce nationally representative estimates and correct standard errors, the complex survey design limits the applicability of some traditional model fit indices; multicollinearity and sparse-category instability were assessed and discussed in the Materials and Methods section, but cannot be fully eliminated.

Finally, the NHAMCS outpatient department sample reflects hospital-based outpatient clinics and may not capture outpatient mental healthcare delivered in private office-based practices, community mental health centers, school-based clinics, telehealth platforms, or other non-hospital settings. Therefore, the findings should be interpreted as representative of hospital outpatient visits during 2005-2011 rather than all settings where outpatient mental healthcare occurs. Despite these limitations, the pooled analyses provide valuable national-level insights into demographic disparities in outpatient mental health service utilization for the study period.

## Conclusions

Racial and ethnic disparities were the strongest predictors of outpatient mental health service utilization in this study. Hispanic and “Other” racial groups were significantly less likely to receive care compared to non-Hispanic Whites. These findings highlight the need for targeted efforts to improve equitable access to outpatient mental health services in the United States. Beyond individual-level tailoring, our findings point to broader structural and policy considerations. Structural barriers, including language discordance, shortages of culturally and linguistically concordant providers, geographic maldistribution of mental health professionals, stigma shaped by social context, and socioeconomic determinants of access, are likely contributors to the observed disparities. Addressing these barriers will require multi-level strategies such as increasing the diversity of the behavioral health workforce, expanding language-concordant and interpretation services, integrating behavioral health into primary care and community settings, strengthening insurance coverage and enforcement of mental health parity, and investing in community outreach and education to reduce stigma. Policy efforts should also consider expanding access to telehealth, school- and community-based mental health services, and workforce training in cultural competence and trauma-informed care. Finally, improved national surveillance that includes outpatient, office-based, and community settings, with richer measures of socioeconomic status, language, and illness severity, is needed to monitor progress and inform interventions. Future research should examine longitudinal patterns, the role of social determinants, and the effectiveness of targeted interventions aimed at reducing these inequities.
